# Short-Term Effects of a High Nitrate Diet on Nitrate Metabolism in Healthy Individuals

**DOI:** 10.3390/nu7031906

**Published:** 2015-03-12

**Authors:** Catherine P. Bondonno, Alex H. Liu, Kevin D. Croft, Natalie C. Ward, Ian B. Puddey, Richard J. Woodman, Jonathan M. Hodgson

**Affiliations:** 1 School of Medicine and Pharmacology, University of Western Australia, Perth, WA 6000, Australia; E-Mails: aliu@meddent.uwa.edu.au (A.H.L.); kevin.croft@uwa.edu.au (K.D.C.); natalie.ward@uwa.edu.au (N.C.W.); ian.puddey@uwa.edu.au (I.B.P.); jonathan.hodgson@uwa.edu.au (J.M.H.); 2 Flinders Centre for Epidemiology and Biostatistics, Flinders University, Adelaide, SA 5042, Australia; E-Mail: richard.woodman@flinders.edu.au

**Keywords:** high nitrate diet, nitrate metabolism, nitric oxide

## Abstract

Dietary nitrate, through the enterosalivary nitrate-nitrite-NO pathway, can improve blood pressure and arterial stiffness. How long systemic nitrate and nitrite remain elevated following cessation of high nitrate intake is unknown. In 19 healthy men and women, the time for salivary and plasma nitrate and nitrite to return to baseline after 7 days increased nitrate intake from green leafy vegetables was determined. Salivary and plasma nitrate and nitrite was measured at baseline [D0], end of high nitrate diet [D7], day 9 [+2D], day 14 [+7D] and day 21 [+14D]. Urinary nitrite and nitrate was assessed at D7 and +14D. Increased dietary nitrate for 7 days resulted in a more than fourfold increase in saliva and plasma nitrate and nitrite (*p* < 0.001) measured at [D7]. At [+2D] plasma nitrite and nitrate had returned to baseline while saliva nitrate and nitrite were more than 1.5 times higher than at baseline levels. By [+7D] all metabolites had returned to baseline levels. The pattern of response was similar between men and women. Urinary nitrate and nitrate was sevenfold higher at D7 compared to +14D. These results suggest that daily ingestion of nitrate may be required to maintain the physiological changes associated with high nitrate intake.

## 1. Introduction

A potential candidate for the cardioprotective effect of a vegetable-rich diet is nitrate. Dietary nitrate, found predominantly in green leafy vegetables, elevates systemic nitrite through an enterosalivary pathway. Nitrite has the potential to be reduced to nitric oxide (NO), a molecule that plays a key role in cardiovascular function [1]. Plasma nitrite, in particular, can be readily reduced to NO and is associated with measures of vascular function [2]. Indeed, there is now consistent data showing reduced blood pressure and improved arterial stiffness, with concomitant increases in nitrite, after a single dose of dietary nitrate [3,4]. There is some evidence for a chronic effect although this is not consistent [5,6,7,8,9]. Saliva, plasma and urine nitrate and nitrite derive from dietary nitrate intake as well as NO metabolism [10]. After ingestion of nitrate blood, salivary and urinary nitrate and nitrite concentrations increase. With a single dose of dietary nitrate, these concentrations return to baseline levels within 24 h [11]. The length of time for these concentrations to return to baseline levels after a chronic period of high dietary nitrate intake is undetermined. This may be relevant to dietary advice such as whether regular (daily) ingestion of nitrate-rich food is required to maintain its cardioprotective benefits.

We have previously conducted a randomized controlled cross-over trial comparing the effect of a high nitrate intake (greater than 300 mg/day) from green leafy vegetables with a low nitrate intake (less than 100 mg/day) for 7 days on plasma, salivary and urinary nitrate and nitrite and blood pressure in 38 volunteers and have reported the main primary outcome data [6]. Although the high nitrate diet resulted in significant elevation in systemic nitrate and nitrite we observed no significant effect on blood pressure. For those volunteers randomised to follow the high nitrate diet for the first intervention period (*n* = 19), the collection of saliva, plasma and urine nitrate and nitrite data at baseline, end of high nitrate period, through the washout period and subsequent low nitrate intervention period allowed us to estimate how long these metabolites stay elevated following cessation of a high nitrate diet.

## 2. Method

The methods and design of the overall study have been published previously [6] but are described here in brief, along with the specific aspects relating to the cohort of participants (19 of 38) who had been randomised to the high nitrate diet in the first intervention and, therefore, underwent the extended washout part of the study.

### 2.1. Participants

Healthy men and women were recruited from the Perth general population by newspaper advertisement. These participants were aged 50 to 70 years; were non-smokers, were not diabetic; had a body mass index 21 to 30 kg/m^2^; had systolic blood pressure greater than 120 mm Hg and less than 139 mm Hg; did not use antihypertensive medication; did not use antibacterial mouthwash; and did not have a history of any major illness such as cancer or cardiovascular disease.

### 2.2. Study Design

Nineteen participants followed a 7 day low nitrate lead in diet (nitrate intake less than 100 mg/day by restricting green leafy vegetable intake) after which fasting baseline measurements were taken (day 0 [D0]). They then followed a 7 day high nitrate diet after which fasting measurements were taken (day 7 [D7]). The high nitrate diet involved increased intake of green leafy vegetables. The green leafy vegetables were provided to the participants as frozen spinach (>250 g/day) and fresh green salad vegetables (120 g/day), with the objective of increased nitrate intake of more than 300 mg/day. During two days of the high nitrate period, participants completed a food diary detailing their green leafy vegetable intake. Following the high nitrate diet period, participants then again followed a 14 day low nitrate diet. Measurements were taken at day 9 (2 days post high nitrate [+2D]), day 14 [+7D] and day 21 [+14D]. At all visits a fasting blood sample was taken by venepuncture for analysis of plasma nitrate and nitrite and a 5 min saliva sample was taken for analysis of salivary nitrate and nitrite. A 24 h urine sample for analysis of urinary nitrate and nitrite was performed at 7D and +14D.

The study was carried out in accordance with the Declaration of Helsinki and was approved by the University of Western Australia Human Research Ethics Committee. Participants provided written informed consent before inclusion in the study. The trial was registered with the Australian New Zealand Clinical Trials Registry (ACTRN12611000609954).

### 2.3. Nitrate and Nitrite Analysis

Concentrations of nitrate and nitrite in spinach, saliva, plasma and urine samples were determined in frozen samples using gas chromatography-mass spectrophotometry (GC-MS) [12]. Nitrate content of the fresh green leafy vegetables provided for the high nitrate diet intervention were estimated using mean literature values [13]. Estimates were based on the proportional weights of specific types of leafy green vegetables included in the salad mix. The frozen spinach and fresh green leafy vegetables provided to the participants contained 0.9 mg/g and an estimated 1 mg/g nitrate respectively. Nitrite contamination in prefilled EDTA blood collection tubes was determined and subtracted from the final plasma nitrite concentration.

### 2.4. Statistics

Statistical analyses were performed using SPSS 21.0 (SPSS Inc, Chicago, IL). Urinary nitrate and nitrite was assessed using paired *t*-tests. Plasma and salivary nitrate and nitrite were assessed using repeated measures ANOVA. The within-subject covariance structure was not modelled. Each day was compared with D0. Bonferroni’s adjustment was used for multiple comparisons. Results in the text and table are presented as mean ± SD. A two-tailed type-1 error rate of *p* < 0.05 was the level of significance used for all hypothesis testing.

## 3. Results

### 3.1. Participants and Dietary Nitrate Intake

A total of nineteen participants (7 men and 12 women) completed the study. During the 7 day high nitrate diet, participants increased their dietary nitrate intake from frozen spinach and fresh green leafy salad vegetables by 402 ± 86 mg/day (Range: 225 to 570 mg/day). Eighteen of the 19 participants increased their dietary nitrate intake by more than 300 mg/day.

### 3.2. Saliva, Plasma and Urine Nitrate and Nitrite

Saliva, plasma and urine nitrate and nitrite values at baseline [D0]; end of the 7 day high nitrate diet [D7] and 2 [+2D], 7 [+7D] and 14 [+14D] days after the end of the high nitrate diet are presented in Table 1.

**Table 1:** Saliva, plasma and urine nitrate and nitrite values at baseline [D0], end of the 7 day high nitrate diet [D7] and 2 [+2D], 7 [+7D] and 14 [+14D] days after the end of the high nitrate diet ^a^.

Saliva/Plasma/Urine	Nitrate/nitrite	D0	D7	+2D	+7D	+14D
Saliva ^b^	Nitrate μmol/L	246.3 (174.8)	1437.8 ** (706.5)	593.2 * (477.7)	322.4 (270.0)	238.3 (143.7)
	Nitrite μmol/L	90.6 (50.9)	532.4 ** (219.3)	191.3 (160.4)	100.2 (66.6)	89.0 (53.4)
Plasma ^b^	Nitrate μmol/L	24.8 (11.9)	154.8 ** (66.0)	36.7 (26.3)	25.0 (13.9)	24.2 (9.2)
	Nitrite μmol/L	2.0 (1.6)	8.3 ** (7.3)	2.8 (2.3)	2.1 (2.2)	1.9 (1.4)
Urine ^c^	Nitrate μmol/day		2985.7 (1250.4)			427.7 ** (100.8)
	Nitrite μmol/day		865.7 (457.5)			102.0 ** (96.2)

^a^ All values are presented as mean (SD); ^b^ Effects were analysed using repeated measures ANOVA in SPSS. Bonferroni’s adjustment was used for multiple comparisons; ^c^ Effects were analysed using paired *t*-tests in SPSS; *p < 0.05; ** *p* < 0.001.

**Figure 1:**
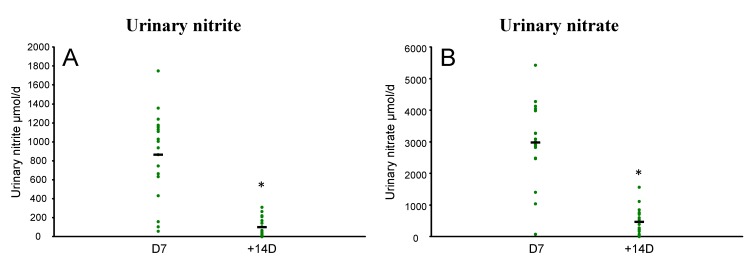
Effect of 7 day high nitrate diet in healthy men and women (*n* = 19) on (**A**) urine nitrite and (**B**) urine nitrate measured at the end of the 7 day period [D7] and 14 days [+14D] after the end of the high nitrate diet for each participant (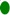), and mean (-). Effects were analysed using paired *t*-tests in SPSS (* *p* < 0.001 compared to D7).

At +14D urine nitrate and nitrite concentrations were significantly lower (*p* < 0.001,) by more than 700%, compared to concentrations at the end of the 7 day high nitrate diet [D7] (Figure 1). Urinary nitrate concentrations were significantly higher for men compared to women at both D7 and +14D (*p* < 0.05).

**Figure 2:**
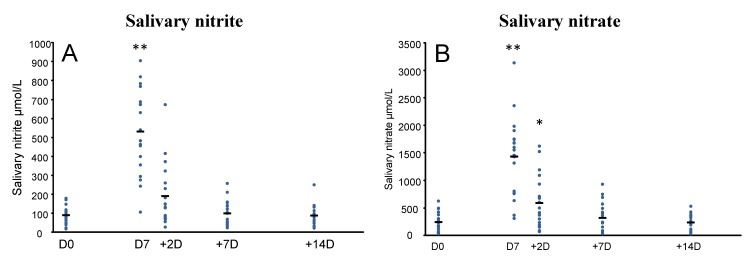

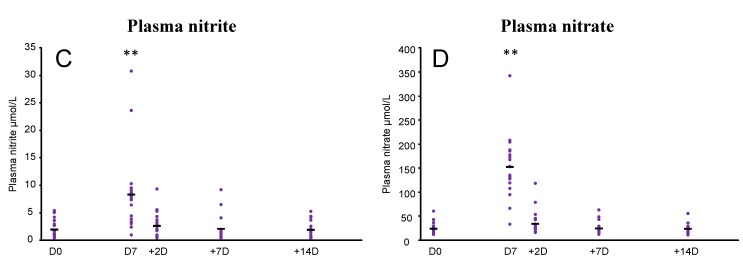
Effect of 7 day high nitrate diet in healthy men and women (*n* = 19) on (**A**) salivary nitrite; (**B**) salivary nitrate; (**C**) plasma nitrite and (**D**) plasma nitrate concentrations, measured at baseline [D0], at the end of the 7 day period [D7], and 2 [+2D], 7 [+7D] and 14 days [+14D] after the end of the high nitrate diet for each participant (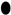), and mean (-). Effects were analysed using repeated measures ANOVA in SPSS (* *p* < 0.05; ** *p* < 0.001 compared to D0). Bonferroni’s adjustment was used for multiple comparisons.

Compared to D0, saliva and plasma nitrate and nitrite concentrations were significantly increased (*p* < 0.001) at D7. The high nitrate diet resulted in a 5- and 6-fold increase in salivary nitrate and salivary nitrite respectively, a 6- and 4 -fold increase in plasma nitrate and plasma nitrite respectively. At +2D, saliva nitrate had decreased but was still significantly elevated compared to D0 (*p* = 0.02). However, salivary nitrite (*p* = 0.08), plasma nitrate (*p* = 1.0) and plasma nitrite (*p* = 0.5) were no longer higher than at D0. By +7D and +14D all metabolites had returned to D0 (baseline) levels (Figure 2). The pattern of saliva and plasma nitrate and nitrite response over the 21 days was similar between men and women.

## 4. Discussion

Following a shift from 7 days of high dietary nitrate from green leafy vegetables to low nitrate intake, saliva and plasma nitrate and nitrite had reduced by 2 days and returned to basal concentrations by 7 days after the end of the high nitrate diet. Plasma nitrite, in particular, was no longer elevated 2 days post the high nitrate period. Our data supports the idea that the benefits of chronic high nitrate intake on blood pressure and arterial stiffness observed in clinical trials may largely be due to continual acute effects on molecules with the potential to be converted to NO. Regular (daily) ingestion of nitrate-rich food may be required to maintain the cardioprotective benefits observed with high dietary nitrate intake.

The two major sources of endogenous nitrate and nitrite are as end products NO metabolism and the diet. Nitrate and nitrite formed as end products of NO metabolism have the potential, through the enterosalivary nitrate-nitrite-NO pathway, to be recycled back into bioactive NO [14]. Through this same pathway dietary nitrate intake can augment endogenous levels of nitrite and therefore, potentially, NO [14]. Vegetable consumption accounts for 80% of dietary nitrate intake [15] and while there is some nitrite intake from the diet, the majority is derived from endogenous biochemical pathways. After dietary nitrate ingestion and effective absorption in the gastro-intestinal tract, plasma nitrate concentrations increase. Approximately one-quarter of plasma nitrate, is actively absorbed by the salivary glands and concentrated by a factor of 10 in saliva compared to plasma nitrate [10]. As a tenfold increase in salivary nitrate compared to plasma nitrate was observed after 7 days of high nitrate intake in the current study, our results are consistent with this estimate. Two days post the end of the high nitrate intake plasma nitrate had returned to baseline levels but salivary nitrate was still slightly elevated. Nitrate in the saliva is reduced to nitrite by nitrate reducing bacteria located in deep clefts on the dorsal surface of the tongue. A fivefold increase in salivary nitrite concentrations was observed at day 7. This increase had attenuated but was still double two days post the high nitrate diet. When swallowed, part of the salivary nitrite is immediately protonated to form nitrous acid in the acidic conditions of the stomach. Nitrous acid decomposes to NO and other nitrogen oxides. The salivary nitrite that escapes the gastric conversion to NO enters the systemic circulation. A fourfold increase in plasma nitrite was observed at day 7 and this had returned to baseline by two days post the high nitrate diet. How nitrite is reduced to NO is unclear but improvements in blood pressure and arterial stiffness are seen with concomitant increases in plasma nitrite after dietary nitrate intake [3,4]. Indeed, plasma nitrite, through its bioactivation to NO, is now recognised to be a critical pathway regulating basal vascular tone, arterial stiffness and, therefore, blood pressure [16].

The half-lives of nitrate, nitrite and NO are 5–8 h, 20–45 min and 1–2 ms respectively [17,18]. Acute studies with a single dose of dietary nitrate have observed a return to baseline of saliva and plasma nitrate and nitrite within 24 h [11,19]. In the current study, two days post the end of the high nitrate diet salivary nitrate and nitrite, although decreased compared to day 7 of the high nitrate diet, were still elevated compared to baseline levels. Plasma nitrate and nitrite has returned to baseline levels. To our knowledge, there is only one other study that has examined markers of nitrate metabolism after chronic dietary nitrate intake. Kapil et al recently observed that salivary, plasma and urinary nitrate and nitrite had returned to baseline levels 2 weeks after a 4 week supplementation with beetroot juice in hypertensive individuals [9]. The decrease in blood pressure observed with high nitrate supplementation was reversed at the end of the 2 week washout. No measurements were performed during the 2 week washout.

Evidence suggests that as much as 25% of endogenously produced and dietary derived nitrate is recycled through the enterosalivary nitrate-nitrite pathway [20]. Approximately 70% of nitrate is excreted in urine within 48 h [21]. Another source of nitrate excretion is sweat. Urinary nitrate and nitrite excretion at day 7 post the high nitrate diet was sevenfold higher compared to 14 days post the end of the high nitrate diet. The observed increase in urinary nitrate is consistent with other studies. While it has been suggested that nitrite is not detected in urine under normal physiological conditions and indicates urinary tract infection, urinary nitrite was detected in the current study and has been reported in other studies [14,22,23]. The increase in urinary nitrite levels at day 7 compared to 14 days post the end of the high nitrate diet, however, is higher than previously observed.

As nitrate and nitrite are end-products of NO metabolism and, through the enterosalivary nitrate-nitrite-NO pathway, have the potential to be converted to bioactive NO, the focus in the literature has been on quantifying these molecules as indicators of NO status. NO’s short half-life, gaseous nature and free radical structure makes direct measurement of NO an analytical challenge. Using circulating nitrate and nitrite as a direct marker of NO bioactivity, in the absence of and after dietary nitrate intake however, is not without controversy. This is due to an incomplete understanding of the biology of NO, nitrite and nitrate; different analytical approaches as well as possible laboratory sources of nitrite contamination. Increases and decreases in present study indicate changes in metabolites with the potential to be converted to NO. They cannot, however, be taken as a direct measurement of NO bioavailability. We have not also explored other possible reactive nitrogen oxides, such as *S*-nitrosothiols, in plasma or tissues that may act as a source of NO. It is possible that these remain elevated while plasma nitrite levels return to baseline levels.

While the values in the present study fall within the range of those observed in other studies, due to different analytical approaches it is impossible to make a direct comparison. The nitrite levels in this study are clearly higher (approximately 10-fold) compared to values measured by gas-phase chemiluminescence, a reference method [3]. Despite this the data shows elevation due to nitrate loading and change over time.

In conclusion, these results indicate that the increase in systemic nitrate and nitrite after 7 days of high nitrate intake are decreased by day 2 and are returned to baseline levels by day 7 after cessation of nitrate intake. As these compounds, through the enterosalivary nitrate-nitrite-NO pathway, have the potential to form bioactive NO this data suggests that daily ingestion of nitrate may be required to maintain the cardioprotective benefits associated with high nitrate intake.
